# 

**Published:** 1963-04

**Authors:** 


					THE
Bristol medico-chirurgical journal
(Incorporating the Medical Journal of the South-West)
Established 1883
V?L- 78 (ii) April 1963 no. 288
PUBLISHED QUARTERLY
ANNUAL SUBSCRIPTION 16/- POST FREE
i
PUBLISHED UNDER THE AUSPICES OF THE
BRISTOL MEDICO-CHIRURGICALSOCIETY
Editorial Committee
Dr. N. J. Brown, Southmead Hospital, Bristol. Joint Editor.
Dr. A. B. Raper, Department of Pathology, Bristol Royal Infirmary.
Joint Editor.
Dr. J. Macrae, Ham Green Hospital.
Dr. R. C. Wofinden, Central Clinic, Bristol.
Mr. A. E. S. Roberts, Medical Library, University of Bristol. Secretary.
Local Correspondents
Bath . . Mr. A. D. Bateman, 3 The Circus, Bath.
Exeter . . Dr. L. N. Jackson, Mount Jocelyn, Crediton, Devon.
Gloucester. Dr. R. F. Jarrett, 9 College Green, Gloucester.
Plymouth . Dr. C. R. Croft, Lexden, Hartley Av., Plymouth.
Taunton . Dr. M. McAlley, i The Crescent, Taunton.
Torquay . Dr. A. Robinson Thomas, 20 Devon Square, Newton Abbot, Devon.
Truro . . Dr. F. D. M. Hocking, St. Andrews, Perranporth, Cornwall.
Yeovil. . Mr. J. A. Vere Nicoll, Knapp House, Yeovil, Somerset.
NOTICE TO CONTRIBUTORS
Original articles are invited, provided they have not been published elsewhere. Notes
of forthcoming events, meetings held, degrees conferred, staff appointments, and other
local news are welcomed. The editorial committee reserves the right to make literary
corrections.
Illustrations should always be supplied ready for reproduction. All re-touchings ot
other operations required will be charged to the author. Line drawings should be drawn
in Indian ink. We regret that we must ask authors to pay for making blocks, if this Is
necessary.
J. W. ARROWSMITH LTD., WINTERSTOKE ROAD,
BRISTOL.
Notes and News
Dr. M. D. Turner has been appointed associate professor of medicine in the Universiy
Rochester Medical School.
Mr. R. D. Stride has been appointed consultant E.N.T. surgeon to the Macclesfield and
district and South Cheshire hospital groups.
Examination Results
University of Bristol M.D.: G. W. Manley, R. K. M. Sanders.
M.R.C.P.: I. Surveyor, B. P. Harrold.
PRIZE ESSAYS, UNIVERSITY OF BRISTOL
Russell Cooper Prize: Miss H. B. Jones
Francis Dudley Memorial Prize: J. F. Morris.
64
I
\
J. W. Arrowsmith I

				

